# Coronavirus Disease 2019 (COVID-19) Lockdown: Morbidity, Perception, Behaviors, and Attitudes in French Families From the PARIS Birth Cohort

**DOI:** 10.3389/fpubh.2022.907456

**Published:** 2022-05-24

**Authors:** Antoine Citerne, Fanny Rancière, Célina Roda, Isabelle Momas

**Affiliations:** ^1^Health Environmental Risk Assessment (HERA) Team, Centre of Research in Epidemiology and Statistics (CRESS), Inserm, National Research Institute for Agriculture, Food and the Environment (INRAE), Université de Paris, Paris, France; ^2^Faculté de Pharmacie de Paris, Université de Paris, Paris, France; ^3^Cellule Cohorte, Direction de l'Action Sociale de l'Enfance et de la Santé, Mairie de Paris, Paris, France

**Keywords:** adolescents, birth cohort, cluster analysis, COVID-19, household transmission, preventive measures, stress, COVID-19 lockdown

## Abstract

**Background:**

Few studies have examined the overall experience of adolescents and their families during COVID-19 lockdowns. This study describes COVID-19-related morbidity in the PARIS birth cohort families during the first lockdown in France and identifies family profiles in terms of morbidity, perception, behaviors, and attitudes.

**Methods:**

Online questionnaires were sent to adolescents of the PARIS birth cohort and their parents. Possible COVID-19 was defined by symptoms using the ECDC definition. Household transmission was estimated by calculating the observed clinical secondary attack rates. Perception, behaviors and attitudes were assessed by levels of stress, degree of satisfaction regarding levels of information about COVID-19, degree of agreement with the lockdown and preventive measures. COVID-19 morbidity in adolescents and parents was compared using chi-squared or Student's *t*-tests. Within each family, perception, behaviors, and attitudes were compared between adolescents and parents using matched-pairs tests. To identify contrasting family profiles, a K-means cluster analysis was implemented.

**Results:**

Of 1,549 families contacted, 1,051 (68%) participated. Adolescents were less affected by possible COVID-19 than their parents (138.7 vs. 192.7 per 1,00,000 person-days). Household transmission of possible COVID-19 was higher when possible COVID-19 came from adults than from adolescents. Most families implemented preventive measures. Adolescents and parents generally shared the same attitudes, but adolescents were less compliant with restrictive measures. Four family profiles were identified which differed mainly regarding family stress, COVID-19 in the household, and compliance with preventive measures.

**Conclusion:**

Improving information dissemination to parents and adolescents, including dedicated adolescent messages, would increase adherence to preventive measures.

## Introduction

After a cluster of pneumonia cases was reported in Wuhan, China, in December 2019, coronavirus disease 2019 (COVID-19), caused by severe acute respiratory syndrome coronavirus 2 (SARS-CoV-2), was characterized by the World Health Organization as a pandemic on 03/11/2020 (1). At this time, due to the high demand and shortage of reagents, many national health authorities had to reserve tests for people with severe symptoms of COVID-19 or specific situations. Consequently, very few studies have been able to assess the prevalence or incidence of COVID-19 in general population. One solution was to rely on symptoms of COVID-19 (2, 3), or use SARS-CoV-2 serological tests (4). In France, there were studies in specific populations: in socially deprived neighborhoods (5, 6), suburban households (7) or in day-care centers (8); but few focused on adolescents and their families. Furthermore, the susceptibility of adolescents to SARS-CoV-2 infection seems to be similar to adults to date (9–11) but the role of adolescents in the household transmission of the SARS-CoV-2 during lockdown remains unclear. Some studies showed no difference in SARS-CoV-2 transmission between children and adults in households (12, 13), while others showed a lower household transmission rate from children compared to adults (14, 15).

To slow the spread of COVID-19, several countries successively opted for lockdown measures. In France, a lockdown procedure was implemented between 03/17/2020 and 05/10/2020. This national lockdown included: closing public spaces, businesses, services, and schools; restricting travel (except for necessary food shopping, medical care, legal obligations, and work when telecommuting was not possible) and limiting outside time to the vicinity of homes (individual sports activity, dog hygiene). This unprecedented situation led to a sudden disruption in the daily lives of adolescents and their families. In order to assess the impact of these public health measures, it is important to better understand the perception and compliance with these measures. Studying the behaviors and attitudes of adolescents and their families is essential to a more accurate comprehension of the dynamics of transmission during the lockdown and the impact of these measures on adolescents. To our knowledge, no studies considered the overall experience of adolescents and their families in terms of morbidity, perception, behaviors, and attitudes during this exceptional event.

Our aims were, i) to describe the morbidity related to COVID-19 in the families of the PARIS birth cohort; ii) to identify family profiles in terms of morbidity, perception, behaviors, and attitudes, during the COVID-19 pandemic in France from 03/17/2020 to 05/10/2020.

## Methods

### Study Design and Participants

This cross-sectional study was carried-out in the PARIS population-based birth cohort which is composed of healthy newborns living in the Paris area recruited between 2003 and 2006 in five Paris hospitals (16). At the end of the first lockdown in France, 1,549 families with an available email address were invited to participate in a specific survey based on both adolescent and parent online self-administered questionnaires. The present study deals with 1,051 families who answered at least one questionnaire (adolescent and/or parents). The response rate was 68%. The French Ethics Committees approved the PARIS cohort follow-up (permission nos. 031153, 051289; ID-RCB, 2009-A00824-53). Parents and adolescents gave their informed consent.

### Data Collection

Data from online questionnaires included questions on socio-demographic, family, and home characteristics, reported COVID-19 morbidity, perception, behaviors, and attitudes during the first lockdown (03/17/2020 to 05/10/2020).

### Reported COVID-19 Morbidity and Household Transmission

COVID-19 morbidity was assessed for the adolescent, the responding parent, and all relatives in the same household during the lockdown. Families reported symptoms suggestive of COVID-19 since the beginning of the year, date of onset and end of symptoms, potential close contact with a COVID-19-like symptoms case and/or a test-confirmed COVID-19 case in the 14 days prior to symptom onset, doctor-diagnosed COVID-19, test-confirmed COVID-19, medical care, and medications. Possible COVID-19 cases were defined according to the European Centre for Disease Prevention and Control as any person with at least one of the following symptoms: cough, fever, shortness of breath, anosmia, ageusia or dysgeusia (17). Possible COVID-19 cases or test-confirmed COVID-19 cases that occurred during the lockdown period between 03/17/2020 and 05/10/2020 inclusive, were identified within each household. Secondary cases were defined as possible COVID-19 cases that occurred between 2 to 14 days after a primary case from the same household. A household contact of a primary case was defined as a family member or a close relative living in the same household during the full lockdown period who did not develop possible COVID-19 or had test-confirmed COVID-19 before the primary case.

### Perception, Behaviors, and Attitudes

Adolescents and parents assessed their levels of stress (overall and SARS-CoV-2-related) from the beginning of the lockdown on a 0-to-10 scale and their degree of satisfaction regarding the level of information received about SARS-CoV-2. Adolescents reported their degree of agreement with lockdown measures while their parents indicated adolescents' tolerance of the lockdown (0-to-10 scale). Concerning changes in family behaviors during the lockdown, we collected adolescents' and parents' information on potential implementation and reinforcement of COVID-19 preventive measures, as well as the frequencies per week of leaving the house for fresh air or shopping and the number of people they had met the previous day. Finally, the parent questionnaire explored the main reason why respondents changed their behavior during the lockdown. Questionnaires were pilot tested.

### Statistical Analysis

The main characteristics of responding and non-responding families were compared using chi-squared tests. Family and home characteristics during the lockdown were described. Incidence rates of possible COVID-19 were calculated for adolescents and parents during the lockdown (from 03/17/2020 to 05/10/2020 inclusive). For each participant never having contracted a possible COVID-19 before the start of the lockdown, the time-at-risk was calculated. This was the time from the first day of the lockdown to: i) the day when the first symptoms were reported in possible COVID-19 cases; ii) the last day of the lockdown or the day the questionnaire was filled in (if before the end of the lockdown) when no possible COVID-19 was detected. The 95% confidence intervals (CI) were determined using the quadratic approximation to the Poisson log likelihood for the log-rate parameter. The observed clinical secondary attack rates (SAR) were estimated as the proportion of secondary cases among all household contacts and according to the age of the primary case. Among possible COVID-19 cases, adolescents and parents were compared regarding symptoms, clinical characteristics and medical care using chi-squared, Fisher's exact tests and Student's *t*-tests. Within each family, perception, behaviors, and attitudes were compared between adolescents and parents using McNemar and Wilcoxon matched-pairs signed-rank tests. To identify contrasting family profiles during the lockdown, a K-means partitioning cluster analysis was implemented on 70 variables related to socio-demographics, family and home characteristics, COVID-19 morbidity, perception, behaviors, and attitudes. Quantitative variables were standardized using Z-scores. The algorithm was performed over 10,000 iterations and repeatedly fitted with 2 to 10 clusters. The optimal classification was chosen based on Calinski-Harabasz criterion and relevance. To compare family profiles and detect the most discriminating variables, chi-squared, Fisher's exact, and Kruskal-Wallis tests were realized with *post-hoc* Tukey's HSD (honestly significant difference) test. All analyses were performed on Stata/SE, using the complete-case method.

## Results

### Participants

Of the 1,549 families to whom questionnaires were sent, 866 adolescents (13–17 years old) and 966 parents responded (Supplementary Figure 1). No differences were observed between participating and non-participating families for sex of the adolescent, place of residence at birth, parental socioeconomic status (SES) and presence of older siblings (Supplementary Table 1).

### Family and Home Characteristics

Table 1 presents the family and home characteristics during the lockdown. Six percent of the adolescents spent most of the lockdown out of their main residence. The median number of persons in the household was four with a median area of 25 m^2^/person. At least one parent had an occupation deemed “essential” (e.g., health care, food manufacture and supply) in 29.9% of the families: 40 percent of this group were health professionals.

**Table 1:** Family and home characteristics of the PARIS birth cohort during the lockdown.

	***n* (%)**
Lockdown in a city of more than 1,00,000 inhabitants, *n* (%)	355 (41.9)
Adolescent place of residence most of the time	
Parents' home only, *n* (%)	731 (85.6)
Alternating homes (separated parents), *n* (%)	73 (8.5)
In a second home, *n* (%)	43 (5.0)
Elsewhere, *n* (%)	8 (0.9)
Number of children in the household (including participants)^*^	
1, *n* (%)	199 (24.0)
2, *n* (%)	420 (50.7)
3, *n* (%)	155 (18.7)
4 or more, *n* (%)	55 (6.6)
Number of adults in the household (including participants)^*^	
1, *n* (%)	86 (10.4)
2, *n* (%)	588 (70.9)
3, *n* (%)	116 (14.0)
4 or more, *n* (%)	39 (4.7)
Housing density	
< one person per room, *n* (%)	378 (45.7)
One person per room, *n* (%)	295 (35.6)
More than one person per room, *n* (%)	155 (18.7)
Adolescent with a shared room, *n* (%)	135 (15.9)
No garden, yard, terrace, or balcony to get fresh air, *n* (%)	120 (14.1)
At least one parent working out of the home, *n* (%)	352 (37.1)

* *Maximum if several households*.

### Reported COVID-19 Morbidity

From 01/01/2020 to 05/10/2020, there were 159 possible COVID-19 cases in adolescents with no difference detected according to sex, age, body mass index and family SES. During lockdown, possible COVID-19 was developed by 53 adolescents and 78 parents for 38,206 and 40,487 person-days-at-risk (Table 2).

**Table 2:** Comparison of prevalence and incidence rates of COVID-19 outcomes between adolescents and parents in the PARIS birth cohort.

	**Adolescents** **(*n* = 832)**	**Parents** **(*n* = 936)**	***p*** -value^*^
**From the beginning of the year to the end of the lockdow*****n*** **(01/01/2020 to 05/10/2020, inclusive)**			
Possible COVID-19 prevalence, *n* (%)	159 (19.1)	218 (23.3)	0.03
Possible COVID-19 and close contact in the 14 days prior to symptom onset with a person with COVID-19-like symptoms, *n* (%)	29 (3.5)	64 (6.8)	<0.001
Possible COVID-19 and close contact in the 14 days prior to symptom onset with a test-confirmed COVID-19 case, *n* (%)	11 (1.3)	23 (2.5)	0.08
Doctor diagnosed COVID-19 prevalence, *n* (%)	17 (2.0)	60 (6.4)	<0.001
Test-confirmed COVID-19 prevalence, *n* (%)	2 (0.2)	12 (1.3)	0.02
**During the lockdow*****n*** **(03/17/2020 to 05/10/2020, inclusive)**			
Possible COVID-19 incidence rate, per 100,000 person-days (95% CI)	138.7 (106.0, 181.6)	192.7 (154.3, 240.5)	0.06

*95% CI, Confidence interval at 95%*.

* *Chi-squared tests and Fisher's exact test were used to compare adolescents' and parents' outcomes*.

Concerning possible COVID-19 cases, fewer adolescents suffered from fatigue and more presented rhinitis-like and gastrointestinal symptoms, compared with parents (Table 3). The median recovery time in adolescents and parents with possible COVID-19 was 9 days (first-third quartile: 5–30 days) and 16 days (7–31 days).

**Table 3:** Comparison of clinical characteristics and medical care between adolescents and parents with possible COVID-19 in the PARIS birth cohort.

	**Adolescents (*n* = 159)**	**Parents (*n* = 218)**	***p*** -value^*^
**Symptoms**			
Fever, *n* (%)	89 (56.0)	118 (54.1)	0.72
Shivers, *n* (%)	49 (30.8)	59 (27.1)	0.43
Muscle or joint pain, *n* (%)	55 (34.6)	82 (37.6)	0.55
Fatigue, *n* (%)	63 (39.6)	122 (56.0)	0.002
Headache, *n* (%)	100 (62.9)	141 (64.7)	0.72
Sore throat, *n* (%)	69 (43.4)	80 (36.7)	0.19
Cough, *n* (%)	98 (61.6)	148 (67.9)	0.21
Shortness of breath, *n* (%)	29 (18.2)	57 (26.2)	0.07
Dyspnea, *n* (%)	35 (22.0)	37 (17.0)	0.22
Chest pain, *n* (%)	27 (17.0)	38 (17.4)	0.91
Phlegm, *n* (%)	12 (7.6)	12 (5.5)	0.42
Rhinorrhea, *n* (%)	82 (51.6)	90 (41.3)	0.05
Nasal congestion, *n* (%)	85 (53.5)	56 (25.7)	<0.001
Sneeze, *n* (%)	81 (50.9)	63 (28.9)	<0.001
Conjunctivitis, *n* (%)	19 (12.0)	14 (6.4)	0.06
Loss of appetite, *n* (%)	36 (22.6)	32 (14.7)	0.05
Nausea, *n* (%)	32 (20.1)	27 (12.4)	0.04
Vomiting, *n* (%)	11 (6.9)	13 (6.0)	0.71
Diarrhea, *n* (%)	34 (21.4)	40 (18.4)	0.46
Abdominal pain, *n* (%)	51 (32.1)	33 (15.1)	<0.001
Anosmia, *n* (%)	20 (12.6)	38 (17.4)	0.20
Ageusia, *n* (%)	27 (17.0)	43 (19.7)	0.50
**Medical care**			
No, *n* (%)	91 (57.2)	97 (44.5)	0.02
Telemedicine, *n* (%)	18 (11.3)	63 (28.9)	<0.001
Community doctor, *n* (%)	36 (22.6)	51 (23.4)	0.86
Emergency consultation, *n* (%)	4 (2.5)	12 (5.5)	0.20
Pharmacist, *n* (%)	5 (3.1)	8 (3.7)	0.78
Other health professional, *n* (%)	12 (7.6)	3 (1.4)	0.003
**Medication intake**			
No, *n* (%)	52 (32.7)	49 (22.5)	0.03
Pain or fever medicines, *n* (%)	84 (52.8)	133 (61.0)	0.11
Cough medicines, *n* (%)	22 (13.8)	26 (11.9)	0.58
Antivirals, *n* (%)	2 (1.3)	1 (0.5)	0.58
Antibiotics, *n* (%)	14 (8.8)	27 (12.4)	0.27
Respiratory or allergic medicines, *n* (%)	6 (3.8)	12 (5.5)	0.47
Homeopathy, *n* (%)	8 (5.0)	6 (2.8)	0.25
Alternative medicine, *n* (%)	11 (6.9)	27 (12.4)	0.08
Other medicines, *n* (%)	5 (3.2)	12 (5.5)	0.28
Hospitalization, *n* (%)	0 (0)	4 (1.8)	0.14

* *Chi-squared tests and Fisher's exact test were used to compare adolescents' and parents' outcomes*.

### Household Transmission

During the lockdown, 422 possible COVID-19 cases were identified in 291 households. The observed clinical SAR of possible COVID-19 was 6.8% (95% CI: 5.2, 8.6) among 900 household contacts. This was 4.3% (95% CI: 0.5, 14.8) from children, 4.4% (95% CI: 2.2, 7.7) from adolescents and 7.8% (95% CI: 5.8, 10.4) from adults (Supplementary Table 2).

### Perception, Behaviors, and Attitudes

**Figure 1:**
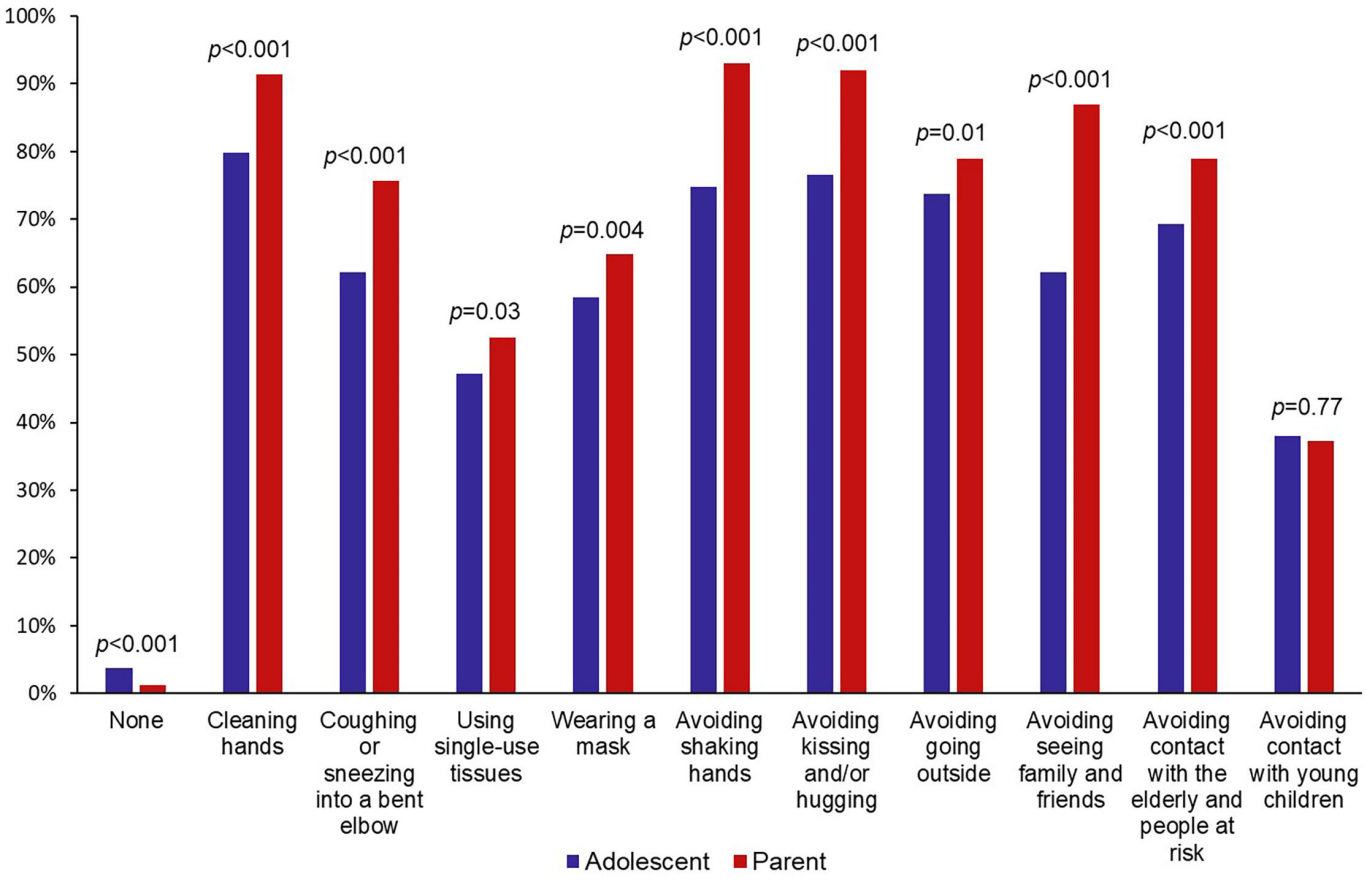
Implementation and reinforcement of COVID-19 preventive measures for adolescents and their parents in the PARIS birth cohort during lockdown.

Adolescents reported general and SARS-CoV-2-related stress levels lower than their parents (*p* < 0.001). Both adolescents and parents used the media as the primary information source regarding SARS-CoV-2 (47.3 vs. 61.1%, *p* < 0.001). Adolescents were more likely to select social networks (11.4 vs. 2.0%, *p* < 0.001) or their relatives and friends (22.5 vs. 0.8%, *p* < 0.001) as primary source of information. Adolescents were fewer to be satisfied with their level of information about the SARS-CoV-2 than their parents (60.0 vs. 66.5%, *p* = 0.006). They tended to be less often satisfied with their level of information when using social networks as their primary source of information (53.1% satisfied using social networks, 61.4% using other sources of information, *p* = 0.12). Concerning behaviors over the whole lockdown, 96.0% of the adolescents implemented or reinforced at least one preventive measure. The preventive measures most frequently cited by adolescents were cleaning hands (79.8%), avoiding kissing and/or hugging (76.6%), avoiding shaking hands (74.8%) and avoiding going outside (73.7%). Compared with their parents, adolescents generally applied significantly fewer preventive measures (Figure 1).

### Family Profiles

A total of 589 families were included in the cluster analysis. Four family profiles were identified. Variables that most distinguished each cluster from another were adolescents' and parents' SARS-CoV-2-related stress levels and overall stress levels, adolescents' tolerance of the lockdown according to the parent, frequency at which the parent left the house for shopping and the number of people the parent had met the previous day (Table 4 and Supplementary Tables 3–7).

**Table 4:** Profiles of families from the PARIS birth cohort based on socio-demographic characteristics, COVID-19 morbidity, perception, behaviors, and attitudes during the lockdown.

***N* = 589**	**Cluster 1 (*n* = 134)**	**Cluster 2 (*n* = 140)**	**Cluster 3 (*n* = 209)**	**Cluster 4 (*n* = 106)**	*p*-value^*^
**Socio-demographic characteristics**					
Lockdown in a city of more than 100,000 inhabitants, *n* (%)	41 (30.6)^c^	60 (42.9)	87 (41.6)	50 (47.2)^c^	0.05
**COVID-19 morbidity**					
Possible COVID-19 in the household					
Adolescent, *n* (%)	18 (13.4)	33 (23.6)	32 (15.3)	23 (21.7)	0.08
**Perception**					
Stress levels from the beginning of lockdown on a scale of 0 to 10					
Overall stress level in adolescents (mean ± SD)	3.5 (2.1)	3.8 (2.4)	3.2 (2.4)^f^	4.2 (2.4)^f^	<0.001
SARS-CoV-2-related stress level in adolescents (mean ± SD)	3.4 (2.3)^b^	3.9 (2.3)^d^, ^e^	2.8 (1.9)^b^, ^d^	3.1 (2.0)^e^	<0.001
Overall stress level in parents (mean ± SD)	4.0 (1.2)^a^, ^b^, ^c^	7.0 (1.0)^a^, ^d^, ^e^	2.3 (1.0)^b^, ^d^, ^f^	6.3 (1.3)^c^, ^e^, ^f^	<0.001
SARS-CoV-2-related stress level in parents (mean ± SD)	6.1 (1.1)^a^, ^b^, ^c^	7.7 (1.1)^a^, ^d^, ^e^	2.5 (1.1)^b^, ^d^, ^f^	3.1 (1.2)^c^, ^e^, ^f^	<0.001
Primary source of information for the adolescent about SARS-CoV-2					
Official information, *n* (%)	29 (24.6)	17 (12.1)	29 (13.9)	23 (21.7)	0.06
Adolescents satisfied with their level of information about SARS-CoV-2, *n* (%)	88 (65.7)^a^	69 (49.3)^a^, ^e^	129 (61.7)	72 (67.9)^e^	0.01
Parents satisfied with their level of information about SARS-CoV-2, *n* (%)	88 (65.7)	85 (60.7)	153 (73.2)	76 (71.7)	0.07
Adolescents' tolerance of lockdown according to the parents, on a scale of 0 to 10 (0: tolerates very badly, 10: tolerates very well).	7.7 (1.5)^b^	7.4 (1.7)^d^	8.2 (1.5)^b^, ^d^, ^f^	7.3 (2.2)^f^	<0.001
**Behaviors**					
Preventive measures against COVID-19 in adolescents					
Avoiding contact with the elderly and people at risk, *n* (%)	101 (75.4)	102 (72.9)	135 (64.6)	81 (76.4)	0.07
Frequency at which adolescents leave home for fresh air or shopping, number per week (mean ± SD)	2.2 (3.2)^c^	2.7 (3.4)	3.2 (4.4)	3.9 (4.7)^c^	0.001
Number of people the adolescent met the previous day, *n* (mean ± SD)	1.6 (3.4)^a^	3.0 (4.2)^a^	2.7 (4.1)	2.9 (4.1)	0.005
Number of people from outside the home seen face to face, number per week (mean ± SD)	0.9 (2.0)	0.8 (1.8)^d^	1.4 (2.6)^d^	0.9 (1.9)	0.07
Preventive measures against COVID-19 in parents					
Cleaning hands, *n* (%)	123 (91.8)	133 (95.0)	183 (87.6)	101 (95.3)	0.04
Coughing or sneezing into a bent elbow, *n* (%)	109 (81.3)	116 (82.9)^d^	148 (70.8)^d^	83 (78.3)	0.03
Avoiding seeing family and friends, *n* (%)	124 (92.5)^b^	124 (88.6)	171 (81.8)^b^	97 (91.5)	0.01
Cleaning the bathroom after each use, *n* (%)	6 (4.5)	8 (5.7)^d^	2 (1.0)^d^	14 (0.9)	0.02
Showering when I get home, *n* (%)	26 (19.4)	35 (25.0)^d^	22 (10.5)^d^	13 (12.3)	0.002
Changing closes when I get home, *n* (%)	26 (19.4)	36 (25.7)^d^	30 (14.4)^d^	17 (16.0)	0.05
Disinfecting everyday objects, *n* (%)	56 (41.8)^b^	60 (42.9)^d^	59 (28.2)^b^, ^d^	29 (27.4)	0.004
Disinfecting door handles, *n* (%)	46 (34.3)	63 (45.0)^d^, ^e^	60 (28.7)^d^	26 (24.5)^e^	0.002
Frequency at which parents leave home for fresh air, number per week (mean ± SD)	2.4 (3.4)	2.6 (3.3)	3.2 (3.6)	3.2 (3.4)	0.03
Frequency at which parents leave home for shopping, number per week (mean ± SD)	1.4 (1.5)^b^, ^c^	1.6 (1.9)^d^, ^e^	2.4 (2.0)^b^, ^d^	2.4 (2.4)^c^, ^e^	<0.001
Number of people the parent met the day before, *n* (mean ± SD)	3.4 (3.7)^a^, ^b^, ^c^	4.1 (4.5)^a^	4.1 (4.4)^b^	4.1 (4.4)^c^	<0.001
**Attitudes**					
Main reason for changing parents' behavior					
Currently with COVID-19, *n* (%)	9 (6.7)^c^	8 (5.7)	4 (1.9)	0 (0)^c^	0.005

*SD, Standard deviation*.

*Variables presented are those that differ with a p < 0.10*.

* *Chi-squared and Kruskal-Wallis tests were used to compare clusters. Post-hoc Tukey HSD (honestly significant difference) comparisons are shown for a p < 0.05 between:*

a *Cluster 2 and Cluster 1;*

b *Cluster 3 and Cluster 1;*

c *Cluster 4 and Cluster 1;*

d *Cluster 3 and Cluster 2;*

e *Cluster 4 and Cluster 2;*

f *Cluster 4 and Cluster 3*.

Cluster 1 (23% of participants) adolescents and parents experienced SARS-CoV-2-related stress, with parents reporting leaving the house for shopping less often than other clusters and meeting fewer people the previous day. Cluster 1 families were less likely to live in a city of more than 1,00,000 inhabitants and were less affected by possible COVID-19 (13.4% of the adolescents).

Cluster 2 (24% of participants) adolescents and parents reported the most SARS-CoV-2-related stress. Parents in Cluster 2 left the house for shopping less often and often applied more extensive preventive measures: cleaning the bathroom after each use, showering, and changing clothes when they arrived home, disinfecting everyday objects and disinfecting door handles. This was the cluster most affected by possible COVID-19 (23.6% of the adolescents).

The largest group was Cluster 3 (35% of participants). Compared to the families from the other clusters, both parents and adolescents showed the least stress and were the least compliant regarding preventive measures. Parents left the house for shopping more frequently. Adolescents saw more people face-to-face and their tolerance of lockdown was the best of all the clusters. The prevalence of possible COVID-19 in Cluster 3 adolescents was 15.3%.

Cluster 4 (18% of participants) adolescents and parents had an overall stress level higher than SARS-CoV-2-related stress level. Families lived more frequently in a city of more than 100,000 inhabitants. Parents were more likely to work outside the home during the lockdown (47.2%) and to leave the house for shopping. Adolescents were less likely to stay at their parents' home for the duration of the lockdown. The prevalence of possible COVID-19 in adolescents in Cluster 4 was 21.7%.

## Discussion

### Key Results

PARIS adolescents were less affected by possible COVID-19 at the end of the first lockdown than their parents. Household transmission during the lockdown was higher when possible COVID-19 came from adults than when it came from adolescents. Most adolescents and parents implemented prevention measures. Nevertheless, adolescents respected these measures less than their parents. Four family profiles were identified. The main factors differentiating these profiles were stress, particularly parental stress, and compliance with preventive measures. This study showed that compliance with preventive measures was strongly related to family stress and COVID-19 morbidity.

### Reported COVID-19 Morbidity

In this study, adolescents were less affected by COVID-19 than their parents. Only the EpiCov study looked at the seroprevalence of SARS-CoV-2 in adolescents in France during the lockdown with a seroprevalence lower in adolescents (3.6%) than in the general population (4.5%) (18). Nevertheless, the prevalence of possible COVID-19 probably led to overestimation of the real prevalence of COVID-19. Our study showed that only 0.2% of the adolescents had test-confirmed COVID-19. This low proportion could be largely explained by the limited access to virological testing during this period. Regarding the clinical manifestations of possible COVID-19 in PARIS teenagers, symptoms were similar to those presented in children studies (19, 20). Adolescents were more likely to develop mild symptoms such as rhinitis-like symptoms and headache. In contrast, parents appeared to develop more severe COVID-19 than adolescents: fatigue symptoms were more frequent, and the duration of symptoms was longer. These results are consistent with previous published findings showing a correlation between age and severity (21, 22). Adolescents suffered from gastrointestinal symptoms more frequently than parents, which has been shown in children (23–25). The household observed clinical SAR of possible COVID-19 (6.8%) was lower than the test-confirmed COVID-19 pooled SAR observed in Thompson et al. (21.1%) and Madewell et al. (16.6%) meta-analysis (26, 27). However, they included few European studies and showed a great heterogeneity of household SAR (0 to 51%) due to different durations of exposure and different testing strategies. The lower SAR observed in our study could be a result of the possible overestimation of primary cases and thus an underestimation of household transmission. However, transmission was lower from adolescents than from adults, which has been previously observed in children but not specifically in adolescents (14, 26, 27). The reasons why adolescents were less affected by COVID-19 than adults could be explained by a greater exposure to SARS-CoV-2 in adults than in adolescents due to the need to go out for shopping or work. Moreover, because the viral load is lower and clearance is faster in the milder COVID-19, this would help explain why transmission from adolescents was lower than from adults (28).

### Perception, Behaviors, and Attitudes

PARIS adolescents had a lower stress level than their parents. This may be due to lower exposure of adolescents to pandemic-related stress. During the lockdown, adolescents were educated at home while parents had additional role demands that could cause psychological stress (29). Despite this, adolescents were less satisfied with their level of received information about the SARS-CoV-2 than their parents. Adolescents' use of social networks compared with their parents could explain this lower level of satisfaction. Indeed, adolescents using social networks as their primary source of information were less often satisfied with it. It has been shown that adolescents are often suspicious of health information from the Internet, but still use it (30). This study found that adolescents were less compliant with preventive measures than their parents. Adolescents could have felt less concerned about the pandemic than their parents. However, PARIS families generally complied with preventive measures. These results agree with the EPIDEMIC project based on adults of the French population (31). Overall, adolescents tolerated the lockdown well, which could be due to their living conditions related to high SES.

### Family Profiles

Clusters revealed the importance of stress and compliance with lockdown measures in differentiating families. Adolescents generally reported attitudes towards lockdown measures similar to their parents, even if they were less compliant. This could be explained by parental practices and parental coping (32). Furthermore, a probable association between stress, COVID-19, and preventive measures has been pointed out. Cluster 2 reported the higher stress level, was the most affected by COVID-19 and strongly respected preventive measures. Conversely, Cluster 3 reported the lower stress level, was the least affected by COVID-19, and was the least compliant with preventive measures. It has been shown that having had COVID-19 may increase compliance with preventive measures (33). Regarding stress levels, fears of infecting others or oneself may increase compliance with preventive measures (34). In Cluster 1, families were relatively stressed but less affected by COVID-19. They lived less frequently in a large city. Cluster 4 families were stressed but not specifically by SARS-CoV-2. They more often lived in a large city and had one parent working outside the home. The economic or job-related situation of these families may have been an important source of stress.

### Strengths and Limitations

It is one of the first studies to consider the complementary views of both adolescents and parents. The use of two simultaneous self-administered questionnaires made it possible to compare morbidity, perception, behaviors and attitudes of adolescents and parents and to collect a large variety of information. The comparison of COVID-19 morbidity between children and adults has been studied, but rarely in adolescents. This study is innovative in comparing perception, behaviors and attitudes between adolescents and their parents. The use of online questionnaires at the end of the lockdown made it possible to cover the entire duration of the lockdown. Finally, the participation rate was satisfactory and remains comparable to other studies (2, 35). The main limitation is the lack of virological confirmation of possible COVID-19 and the risk its misclassification. Moreover, we cannot exclude a recall bias despite the strong awareness, especially via media. Lastly, this study focuses on a specific population. The Parisian families being of a high SES, these findings cannot be generalized to all French families.

## Conclusion

COVID-19 morbidity, stress and preventive measures were inter-related within families during the lockdown. Adolescents and their parents presented similar attitudes towards lockdown measures, even if adolescents were less compliant. This study underscores that media and relatives are a key prevention medium to focus on when informing adolescents. Improving information dissemination to adolescents and parents, including dedicated adolescent messages, would increase adherence to preventive measures.

## Data Availability Statement

The datasets presented in this article are not readily available because the data analysis is still ongoing. Requests to access the datasets should be directed to the corresponding author (CR, celine.roda@u-paris.fr).

## Ethics Statement

The studies involving human participants were reviewed and approved by French Ethic Committee. Written informed consent to participate in this study was provided by the participants' legal guardian/next of kin.

## Author Contributions

AC: methodology, validation, formal analyses, writing—original draft, and visualization. FR and CR: conceptualization, methodology, validation, investigation, writing—review and editing, and supervision. IM: conceptualization, methodology, validation, investigation, writing—review and editing, supervision, project administration, and funding acquisition. All authors contributed to the article and approved the submitted version.

## Funding

This work was supported by the Paris Municipal Department of Social Action, Childhood, and Health (DASES) and the French National Research Agency (ANR) [16-CE36-0007-01].

## Conflict of Interest

The authors declare that the research was conducted in the absence of any commercial or financial relationships that could be construed as a potential conflict of interest.

## Publisher's Note

All claims expressed in this article are solely those of the authors and do not necessarily represent those of their affiliated organizations, or those of the publisher, the editors and the reviewers. Any product that may be evaluated in this article, or claim that may be made by its manufacturer, is not guaranteed or endorsed by the publisher.
