# Supplementary material for: Coronavirus Disease 2019 (COVID-19) Lockdown: Morbidity, Perception, Behaviors, and Attitudes in French Families From the PARIS Birth Cohort
Source: Front Public Health. 2022 May 24;10:907456. doi: 10.3389/fpubh.2022.907456 (PMC9170922; doi:10.3389/fpubh.2022.907456)
Supplement: Supplementary file 1 [file Data_Sheet_1.pdf]

Supplementary Material

1 Supplementary Figure and Tables

1.1 Supplementary Figure

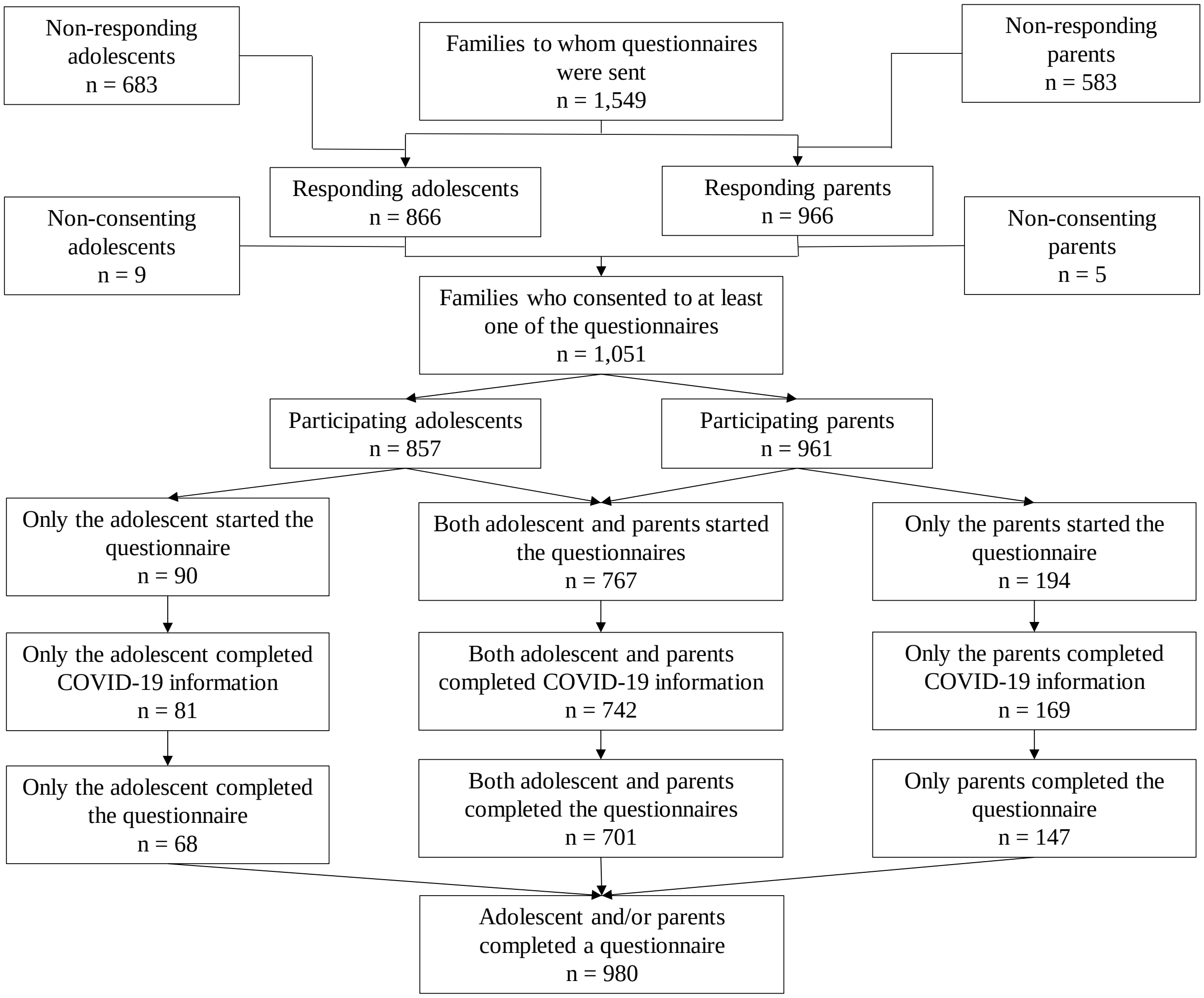

Supplementary Figure 1. Flowchart of the study population.

1.2 Supplementary Tables

Supplementary Table 1. Baseline characteristics of responding and non-responding families from the PARIS birth cohort study.

Baseline characteristics at birth	Responding families (n=1,051)	Non-responding families (n=498)	*p*-value*
Sex of the adolescent			0.27
Female, n (%)	519 (49.4)	231 (46.4)	
Male, n (%)	532 (50.6)	267 (53.6)	
Place of residence at birth			0.15
Paris city, n (%)	640 (60.9)	322 (64.7)	
Paris suburbs, n (%)	411 (39.1)	176 (35.3)	
Mother's socioeconomic status			0.43
Low, n (%)	136 (13.0)	76 (15.3)	
Medium, n (%)	420 (40.0)	198 (39.8)	
High, n (%)	494 (47.0)	223 (44.9)	
Father's socioeconomic status			0.06
Low, n (%)	111 (10.6)	69 (13.9)	
Medium, n (%)	269 (25.7)	138 (27.9)	
High, n (%)	668 (63.7)	288 (58.2)	
Mother's educational level			0.05
Primary, n (%)	6 (0.6)	8 (1.6)	
Secondary, n (%)	90 (8.6)	53 (10.7)	
Post-secondary, n (%)	954 (90.8)	436 (87.7)	
Father's educational level			0.25
Primary, n (%)	9 (0.9)	9 (1.8)	
Secondary, n (%)	154 (14.7)	74 (15.0)	
Post-secondary, n (%)	884 (84.4)	411 (83.2)	
Maternal active smoking during pregnancy			0.57
Yes, n (%)	86 (8.2)	45 (9.0)	
No, n (%)	965 (91.8)	453 (91.0)	
Older sibling(s)			0.96
Yes, n (%)	488 (46.4)	232 (46.6)	
No, n (%)	563 (53.6)	266 (53.4)	

*Chi-squared tests were used to compare participating and non-participating families

Supplementary Table 2. Observed clinical secondary attack rates (SAR) of possible COVID-19 during the lockdown in PARIS birth cohort households.

Population	Primary cases[a]	Secondary cases[b]	Household contact of a primary case[b]	SAR (%)
Total	320	61	900	6.8 (5.2, 8.6)
Children (0-12 years old)	17	2	46	4.3 (0.5, 14.8)
Adolescents (13-17 years old)	87	11	253	4.4 (2.2, 7.7)
Adults (≥18 years old)	203	44	561	7.8 (5.8, 10.4)

Of the 422 possible COVID-19 cases, 320 had complete data. [a] The sum of primary cases by age categories does not equal the total primary cases due to missing data for the ages of 13 subjects. [b] Secondary cases and household contacts of a primary case do not take into account the age of the subjects.

Supplementary Table 3. Clusters' socio-demographic characteristics.

N=589	Cluster 1 (n=134)	Cluster 2 (n=140)	Cluster 3 (n=209)	Cluster 4 (n=106)	*p*-value*
Family socioeconomic status					0.24
Low and medium, n (%)	36 (26.9)	43 (30.7)	61 (29.2)	21 (19.8)	
High, n (%)	98 (73.1)	97 (69.3)	148 (70.8)	85 (80.2)	
At least one parent working out of the home, n (%)	46 (34.3)	47 (33.6)	81 (38.8)	50 (47.2)	0.12
At least one parent in a medical profession, n (%)	14 (10.5)	13 (9.3)	19 (9.1)	15 (14.2)	0.54
Lockdown in a city of more than 100,000 inhabitants, n (%)	41 (30.6)	60 (42.9)	87 (41.6)	50 (47.2)	0.05
Adolescent residence for most of the time					
At my parent(s)' home, n (%)	122 (91.0)	130 (92.9)	191 (91.4)	89 (84.0)	0.10
In a second home, n (%)	5 (3.7)	6 (4.3)	10 (4.8)	9 (8.5)	0.35
Number of children in the household (including participants), maximum if several households					0.74
1, n (%)	27 (20.1)	30 (21.4)	52 (24.9)	26 (24.5)	
2, n (%)	75 (56.0)	73 (52.2)	108 (51.7)	54 (50.9)	
3, n (%)	25 (18.7)	30 (21.4)	36 (17.2)	18 (17.0)	
4 or more	7 (5.2)	7 (5.0)	13 (6.2)	8 (7.6)	
Number of adults in the household (including participants), maximum if several households					0.89
1, n (%)	7 (5.2)	4 (2.8)	6 (2.9)	2 (1.9)	
2, n (%)	104 (77.6)	111 (79.3)	158 (75.6)	78 (73.6)	
3, n (%)	19 (14.2)	19 (13.6)	35 (16.8)	21 (19.8)	
4 or more, n (%)	4 (3.0)	6 (4.3)	10 (4.7)	5 (4.7)	
Housing density					0.58
Less than one person per room, n (%)	67 (50.0)	57 (40.7)	93 (44.5)	47 (44.3)	
One person per room, n (%)	49 (36.6)	58 (41.4)	73 (34.9)	39 (36.8)	
More than one person per room, n (%)	18 (13.4)	25 (17.9)	43 (20.6)	20 (18.9)	
Adolescent with a shared room, n (%)	17 (12.7)	18 (12.9)	37 (17.7)	12 (11.3)	0.35
No garden, yard, terrace or balcony to get fresh air, n (%)	120 (89.6)	126 (90.0)	182 (87.1)	90 (84.9)	0.58
Household with a relative with chronic disease, n (%)	14 (10.5)	9 (6.4)	16 (7.7)	3 (2.8)	0.14
Smoker in the household, n (%)	34 (25.4)	33 (25.6)	51 (24.4)	26 (24.5)	0.99

*Chi-squared and Fisher's exact tests were used to compare clusters

Supplementary Table 4. Clusters' COVID-19 morbidity.

N=589	Cluster 1 (n=134)	Cluster 2 (n=140)	Cluster 3 (n=209)	Cluster 4 (n=106)	*p*-value*
Possible COVID-19 in the household					
Adolescent, n (%)	18 (13.4)	33 (23.6)	32 (15.3)	23 (21.7)	0.08
Parent, n (%)	31 (21.1)	39 (27.9)	42 (20.1)	26 (24.5)	0.41
At least one household member, n (%)	52 (38.8)	71 (50.7)	89 (42.6)	43 (40.6)	0.20
Health problems or death of a relative due to coronavirus, n (%)	6 (4.5)	10 (7.1)	8 (3.8)	8 (7.6)	0.39

*Chi-squared were used to compare clusters

Supplementary Table 5. Clusters' perception during the lockdown

N=589	Cluster 1 (n=134)	Cluster 2 (n=140)	Cluster 3 (n=209)	Cluster 4 (n=106)	*p*-value*
Stress levels from the beginning of the lockdown on a scale of 0 to 10					
Overall stress level in adolescents (mean ± SD)	3.5 (2.1)	3.8 (2.4)	3.2 (2.4)	4.2 (2.4)	<0.001
Coronavirus-related stress level in adolescents (mean ± SD)	3.4 (2.3)	3.9 (2.3)	2.8 (1.9)	3.1 (2.0)	<0.001
Overall stress level in parents (mean ± SD)	4.0 (1.2)	7.0 (1.0)	2.3 (1.0)	6.3 (1.3)	<0.001
Coronavirus-related stress level in parents (mean ± SD)	6.1 (1.1)	7.7 (1.1)	2.5 (1.1)	3.1 (1.2)	<0.001
Primary source of information for the adolescent about coronavirus					
Official information, n (%)	29 (24.6)	17 (12.1)	29 (13.9)	23 (21.7)	0.06
Social networks, n (%)	11 (8.2)	17 (12.1)	25 (12.0)	11 (10.4)	0.68
Adolescents satisfied with the level of information received about coronavirus, n (%)	88 (65.7)	69 (49.3)	129 (61.7)	72 (67.9)	0.01
Primary source of information for the parent about coronavirus					
Official information, n (%)	33 (24.6)	37 (26.4)	51 (24.4)	30 (28.3)	0.88
Information available on social networks, n (%)	11 (8.2)	17 (12.1)	25 (12.0)	11 (10.4)	0.68
Parents satisfied with the level of information received about coronavirus, n (%)	88 (65.7)	85 (60.7)	153 (73.2)	76 (71.7)	0.07
Adolescents in agreement with lockdown measures, n (%)	118 (88.1)	121 (86.4)	181 (86.6)	93 (87.7)	0.97
Adolescents' tolerance of lockdown according to the parents, on a scale of 0 to 10 (0: tolerates very badly, 10: tolerates very well)	7.7 (1.5)	7.4 (1.7)	8.2 (1.5)	7.3 (2.2)	<0.001

Definition of abbreviation: SD = Standard deviation.
*Chi-squared and Kruskal-Wallis tests were used to compare clusters

Supplementary Table 6. Clusters' behaviors during the lockdown

N=589	Cluster 1 (n=134)	Cluster 2 (n=140)	Cluster 3 (n=209)	Cluster 4 (n=106)	*p*-value*
Preventive measures against COVID-19 in adolescents					
None, n (%)	5 (3.7)	3 (2.1)	11 (5.3)	1 (0.9)	0.21
Cleaning hands, n (%)	109 (81.3)	109 (77.9)	172 (82.3)	86 (81.1)	0.77
Coughing or sneezing into a bent elbow, n (%)	87 (64.9)	93 (66.4)	129 (61.7)	68 (64.2)	0.83
Using single-use tissues, n (%)	64 (47.8)	77 (55.0)	92 (44.0)	47 (44.3)	0.20
Wearing a mask, n (%)	82 (61.2)	87 (62.1)	114 (54.6)	65 (61.3)	0.43
Avoiding shaking hands, n (%)	107 (79.9)	111 (79.3)	150 (71.8)	79 (74.5)	0.25
Avoiding kissing and/or hugging, n (%)	106 (79.1)	117 (83.6)	154 (73.7)	78 (73.6)	0.12
Avoiding going outside, n (%)	106 (79.1)	105 (75.0)	156 (74.6)	71 (67.0)	0.20
Avoiding seeing family and friends, n (%)	90 (67.2)	89 (63.6)	123 (58.9)	67 (63.2)	0.47
Avoiding contact with the elderly and people at risk, n (%)	101 (75.4)	102 (72.9)	135 (64.6)	81 (76.4)	0.07
Avoiding contact with young children, n (%)	55 (41.0)	61 (43.6)	81 (38.8)	39 (36.8)	0.71
Frequency at which adolescents leave home for fresh air or shopping, number per week (mean ± SD)	2.2 (3.2)	2.7 (3.4)	3.2 (4.4)	3.9 (4.7)	0.001
Number of people the adolescent met the day before, n (mean ± SD)	1.6 (3.4)	3.0 (4.2)	2.7 (4.1)	2.9 (4.1)	0.005
Number of people from outside the home seen face to face, number per week (mean ± SD)	0.9 (2.0)	0.8 (1.8)	1.4 (2.6)	0.9 (1.9)	0.07
Adolescent involved in an association, n (%)	15 (11.2)	7 (5.0)	11 (5.3)	9 (8.5)	0.13
Preventive measures against COVID-19 in parents					
None, n (%)	0 (0)	0 (0)	0 (0)	0 (0)	N/A
Cleaning hands, n (%)	123 (91.8)	133 (95.0)	183 (87.6)	101 (95.3)	0.04
Coughing or sneezing into a bent elbow, n (%)	109 (81.3)	116 (82.9)	148 (70.8)	83 (78.3)	0.03
Using single-use tissues, n (%)	79 (59.0)	78 (55.7)	97 (46.4)	56 (52.8)	0.12
Wearing a mask, n (%)	82 (61.2)	98 (70.0)	125 (59.8)	69 (65.1)	0.24
Avoiding shaking hands, n (%)	124 (92.5)	129 (92.1)	195 (93.3)	99 (93.4)	0.97
Avoiding kissing and/or hugging, n (%)	126 (94.0)	124 (88.6)	190 (90.9)	100 (94.3)	0.27
Avoiding going outside, n (%)	112 (83.6)	116 (82.9)	160 (76.6)	87 (82.1)	0.31
Avoiding seeing family and friends, n (%)	124 (92.5)	124 (88.6)	171 (81.8)	97 (91.5)	0.01
Avoiding contact with the elderly and people at risk, n (%)	115 (85.8)	115 (82.1)	157 (75.1)	85 (80.2)	0.10
Avoiding contact with young children, n (%)	51 (38.1)	53 (37.9)	75 (35.9)	38 (35.9)	0.97
Living in a separate room, n (%)	4 (3.0)	1 (0.7)	1 (0.5)	1 (0.9)	0.21
Not sharing everyday objects, n (%)	10 (7.5)	18 (12.9)	14 (6.7)	6 (5.7)	0.13
Clean the bathroom after each use, n (%)	6 (4.5)	8 (5.7)	2 (1.0)	14 (0.9)	0.02
Showering when I get home, n (%)	26 (19.4)	35 (25.0)	22 (10.5)	13 (12.3)	0.002

Changing clothes when I get home, n (%)	26 (19.4)	36 (25.7)	30 (14.4)	17 (16.0)	0.05
Disinfecting everyday objects, n (%)	56 (41.8)	60 (42.9)	59 (28.2)	29 (27.4)	0.004
Disinfecting door handles, n (%)	46 (34.3)	63 (45.0)	60 (28.7)	26 (24.5)	0.002
Disinfect or quarantine shopping, n (%)	2 (1.5)	1 (0.7)	1 (0.5)	0 (0)	0.65
Frequency at which parents leave home for fresh air. number per week (mean ± SD)	2.4 (3.4)	2.6 (3.3)	3.2 (3.6)	3.2 (3.4)	0.03
Frequency at which parents leave home for shopping, number per week (mean ± SD)	1.4 (1.5)	1.6 (1.9)	2.4 (2.0)	2.4 (2.4)	<0.001
Number of people the parent met the day before, n (mean ± SD)	3.4 (3.7)	4.1 (4.5)	4.1 (4.4)	4.1 (4.4)	<0.001

Definition of abbreviations: SD = Standard deviation; N/A = not applicable.
*Chi-squared, Fisher's exact and Kruskal-Wallis tests were used to compare clusters

Supplementary Table 7. Clusters' attitudes during the lockdown

N=589	Cluster 1 (n=134)	Cluster 2 (n=140)	Cluster 3 (n=209)	Cluster 4 (n=106)	*p*-value*
Main reason for changing parents' behavior					
Currently with COVID-19, n (%)	9 (6.7)	8 (5.7)	4 (1.9)	0 (0)	0.005
Avoiding getting COVID-19, n (%)	118 (88.1)	121 (86.4)	175 (83.7)	86 (81.1)	0.44
Avoiding transmission of COVID-19, n (%)	103 (76.9)	116 (82.9)	174 (83.3)	94 (88.7)	0.12
Having a relative with a chronic disease in the household, n (%)	17 (12.7)	15 (10.7)	21 (10.1)	4 (3.8)	0.09
Having a relative who worked outside the home in the household, n (%)	4 (3.0)	8 (5.7)	12 (5.7)	7 (6.6)	0.56

*Chi-squared and Fisher's exact tests were used to compare clusters
